# Report of a Case of Tubercular Arthritis of the Knee, with Remarks

**Published:** 1900-09

**Authors:** J. W. Henson

**Affiliations:** Richmond, Va. Professor of Anatomy and Demonstrator of Orthopedics in the University College of Medicine, and Surgeon to Virginia Hospital


### REPORT OF A CASE OF TUBERCULAR ARTHRITIS OF THE KNEE, WITH REMARKS.*

By J. W. HENSON, M.D., RICHMOND, VA.,

Professor of Anatomy and Demonstrator of Orthopedics in the University College of Medicine, and Surgeon to Virginia Hospital.

Last spring a negro boy 12 or 13 years old, suffering with tubercular arthritis of the knee of long standing, was put in my care by a brother physician. Suppuration had been going on for some time, and an ugly looking sinus on the inner side of the joint led down to necrosed bone. It was a nice question whether to amputate or trust to the scoop. I decided, for reasons to be presently stated, in favor of the latter. The joint was freely opened by an incision curving downward, the center of the incision sweeping below the patella. This bone was so much diseased that it was deemed wise to remove it. The inner condyle of the femur and inner tuberosity of the tibia were extensively necrosed; the outer condyle and outer tuberosity were also diseased but less extensively.

With a Volkmann spoon everything diseased, as far as could be ascertained, was removed, the joint thoroughly irrigated and the incision closed; but not doing well at the end of five or six days, it was reopened, suspicious tissues scraped away, and free drainage provided. The joint is now ankylosed but well.

When we have in charge a case of articular arthritis, it should be our duty to have in view first, the saving of the life of the patient by early removal of the local trouble (which is a constant menace as long as it exists), and second, the preservation of the limb with

*Read before the Richmond Academy of Medicine and Surgery, Jan. 23, 1900.

as little deformity as possible. In badly damaged tubercular knee joints the surgeon's chief means of attaining the end is excision in the case of the adult; but in children our conservative aim is confronted with a very serious difficulty, for sawing the epiphysis at this time of life is very apt to arrest, or at least materially diminish the growth of the limb. With the aid of the spoon, however, we may remove all of the epiphysis except a shell of sound bone; but as this shell reaches to the original level there is no loss in length of limb, and the hollow will fill with new bone tissue leaving growth to go on as before. If much scraping is done on both bones we shall very likely get a stiff limb but one that will grow. Therefore, in children afflicted with tubercular knee (which must have surgical attention), there are at our disposal for relief but two measures—erasion and amputation. The following, then, becomes an interesting and important question: How far may tubercular disease of the knee progress and yet be amenable to relief by the use of the Volkmann spoon? This presented itself to me when I decided in favor of erasion in the case reported. I desired to see if it were possible, by this means, to permanently stop the progress of disease in a joint that would be almost universally pronounced past relief by any means except amputation.

The success in this case does not, of course, prove that so bad a joint can always be cured thus, but it does show that some may be; and a further test may prove that many, equally as bad, can be relieved, which would be of great significance to the young who fall heir to this affliction.

There is another point of some moment. It is recommended by some authorities that excision be not done before about the twelfth year of age. Neither epiphysis at the knee becomes united to the shaft before the twentieth year. If they are sawed before they are thus joined the limb will be shortened, finally, by the thickness of the two pieces removed plus the amount of growth lost. Therefore, I strongly urge that if it can possibly be avoided by erasion, excision of the knee be not done unless the full stature of the individual be much more nearly attained than it is at the twelfth year, say not before the fifteenth year.

I have made no mention of flexion, because in children this may follow excision. Resection of the hamstring muscles would best be done to prevent it. Whether it be done or not, the knee should be supported by a plaster cast for several months—eight or twelve—and the patient should not bear weight on the limb for that length of time.

